# Expression analysis of innate immunity related genes in the true/field blast resistance gene-mediated defence response

**DOI:** 10.1080/13102818.2014.978664

**Published:** 2014-11-18

**Authors:** Debing Wang, Yonghua Qin, Jingluan Han, Ling Zhang, Xin Xu, Xuequn Liu, Chuntai Wang, Xinqiong Liu

**Affiliations:** ^a^ Hubei Provincial Key Laboratory for Protection and Application of Special Plants in Wuling Area of China, College of Life Science, South-Central University for Nationalities, Wuhan, Hubei, P.R. China; ^b^ College of Life Science, South China Agricultural University, Guangzhou, Guangdong, P.R. China; ^c^ Agricultural Technology Extension and Service Center of Jingzhou City, Jingzhou, Hubei, P.R. China

**Keywords:** rice blast disease, true resistance, field resistance, signalling pathway

## Abstract

Rice blast resistance (*R*) genes-mediated resistance response depends on various resistance-related genes involved in incompatible interactions. In this work, the expression profiles of innate rice immunity related genes were examined in the mediated resistance response of true/field resistance genes. Three sets of rice near-isogenic lines (NILs) were used: the resistant NILs carrying true resistance genes in the genetic background of the susceptible cultivar Nipponbare (NB), NB-Pib, NB-Pizt, NB-Pik and NB-Pita2; NILs bearing field resistance genes *pi21* in the susceptible cultivar Aichiasahi (AA) AA-pi21, Kahei (KHR). The marker gene *OsWRKY45* of salicylic acid (SA) signalling was upregulated in all tested cultivars. And, *JAmyb* (marker gene of jasmonic acid signalling) showed higher upregulation in the resistance lines with nucleotide-binding sites and leucine-rich repeat (NBS-LRR) *R* genes *Pib*, *Pizt*, *Pik*, *Pita2* and *Pikahei* than in NB and KHS. *SalT* of abscisic acid (ABA) signalling may be involved in the *R/Avr* interaction, including *Pizt*, *Pik*, *pi21* and *Pikahei*. However, *SalT* was shown to negatively regulate *Pib/AvrPib* interaction. *OsPR1b* and *PBZ1* were differentially expressed and strongly activated at a later stage by 48 h post-inoculation. Interestingly, there was evidence that *OsPR1b* and *PBZ1* played an important role in the *pi21-*mediated response. It was shown that *OsRAR1* could be upregulated in the true resistance line NB-Pita2 and the field resistance line KHR, while *OsSGT1* and *OsHSP90* could be upregulated in all tested lines. The involvement of these genes illustrated the complexity of the downstream signalling pathways in the mediated resistance response of true/field resistance genes.

## Abbreviations

ABA: abscisic acid
JA: jasmonic acid
NB: Nipponbare
NBS-LRR: nucleotide-binding sites and leucine-rich repeat
NILs: near-isogenic lines
p.i.: post-inoculation
PRs: pathogenesis-related proteins
qRT-PCR: quantitative reverse transcription PCR
QTL: quantitative trait loci
SA: salicylic acid

## Introduction

Caused by the fungal pathogen *Magnaporthe oryzae*, rice blast disease is recognized as one of the most devastating diseases in rice worldwide, posing a dramatic threat to global food supplies.[1] There have been widespread efforts to control this disease through resistant cultivars and fungicides with limited success. The use of disease resistance (*R*) genes, however, has yielded promising results through efficient and environmentally friendly methods.[2] Rice blast resistance can be generally classified into two main types.[3] The first type of resistance is known as true resistance, or complete resistance, and is conferred by a single dominant gene that protects against infection and reproduction in a specific race of *M. oryzae*.[2] The second type of resistance is known as field resistance, or partial resistance, and is race non-specific, mediated by multiple genes or quantitative trait loci (QTLs) and thought to be substantially more resilient.[3]

In recent years, research has identified over 90 major *R* genes of rice blast disease and more than 20 of those *R* genes have been isolated.[4] Among them, 17 *R* genes, *Pib*, *Pi-ta*, *Pik-h*, *Pi2*, *Pi9*, *Piz-t*, *Pi36*, *Pi37*, *Pik-m*, *Pid3*, *Pi5*, *Pit*, *Pbl*, *Pi-sh*, *Pik*, *Pik-p* and *Pia*, belong to a large *R* gene family, which encodes nucleotide-binding sites and leucine-rich repeat (NBS-LRR) proteins; in addition, *Pid-2* encodes receptor-like kinase proteins [5] and the partial resistance gene *pi21* encodes a proline-rich protein.[6]

Extensive research has been reported on innate plant immunity and various defence responses against microbial pathogen attack mediated through multiple signalling pathways. Such defence responses are a key requirement for the induction of a set of pathogenesis-related proteins (PRs). PRs are defined as plant proteins that are induced specifically in pathological or stress situations; the production and accumulation of PRs represent an essential component of the active plant defence repertoire.[7] One of the critical signalling pathways leading to stress response is performed through salicylic acid (SA), which is constitutively present in high levels in rice.[8,9] In addition, a large body of genetic and molecular evidence has shown that jasmonates, including jasmonic acid (JA) and methyl jasmonate, also provide essential and important endogenous signals that are implicated in induced resistance to pathogen infection.[10] Moreover, abscisic acid (ABA) has also been identified as a signalling molecule and has been implicated as a component in PR gene(s) regulation in plants.[11] RAR1, a zinc-binding protein, is another key component involved in diverse R resistance in plants.[12] RAR1 interacts directly with SGT1 (for suppression of the G2 allele of *Skp1*) and HSP90, which are essential for disease resistance mediated by diverse R proteins.[13]

In this study, the expression profiles of innate rice immune system-related genes were investigated using a candidate gene approach for true resistance and field resistance responses to rice blast disease. The tested genes included the following signalling pathway marker genes: *OsWRKY45* of SA, *JAmy* of JA and *SalT* of ABA; *OsPR1b* and *PBZ1* of PR; and *OsRAR1*, *OsSGT1*, *OsHSP90* genes. The development of understanding in the gene network associated with *R* genes will be highly valuable in the elaboration of strategies to promote durable resistance against rice blast and may further illuminate some of the biochemical and cellular mechanisms underlying host resistance.

## Materials and methods

### Plant materials and pathogen inoculation

This study utilized three sets of rice near-isogenic lines (NILs): the resistant NILs carrying true resistance genes in susceptible cultivar Nipponbare (NB), NB-Pib, NB-Pizt, NB-Pik and NB-Pita2; NILs bearing field resistance genes *pi21* in the susceptible cultivar Aichiasahi (AA) AA-pi21, Kahei (KHR). It is documented that the cultivars NB with *Pish*,[14] AA with *Pia* [15] and Kahei with *Pikahei* [16] are partially resistant to blast disease.

Plants were grown in a greenhouse under natural growth conditions for approximately 14 days (till the fourth-leaf stage) and then transplanted to a plant growth chamber (28 °C/25 °C of day/night temperature, 14 h/10 h light/dark regime and 85% relative humidity) for two days before subsequent treatment. The plants were inoculated with a 2 × 10^5^/mL spore suspension of *M. oryzae* race 007 in 0.02% (v/v) Tween 20, and samples were then collected at three time intervals (0 h, 24 h and 48 h) post-inoculation (p.i.). Six individual plants were pooled in a sample and then frozen with liquid nitrogen prior to total RNA preparation. Three independent experiments were included in the analysis.

### RNA extraction and reverse transcription

Total RNA was isolated from a leaf, using a TRIzol reagent (Invitrogen, USA), according to the instructions of the manufacturer, and then treated with RNase-free DNaseI (Promega, USA) to remove contaminated genomic DNA. First-strand cDNA was then generated using the Superscript^TM^ III First-Strand Synthesis System (Invitrogen, USA) for real-time polymerase chain reaction (RT-PCR). For each sample, two separate reverse transcription reactions were combined. All quantitative reverse transcription PCRs (qRT-PCRs) were based on the same pooled cDNA to ensure uniformity in the results.

### Real-time PCR analysis

Gene-specific primers were designed from publicly available nucleotide sequences (Table 1), while the *OsEF1a* gene was expressed as a housekeeping gene to normalize the samples. A qRT-PCR reaction was performed in 20 μL reaction volume as follows: 1 μL of cDNA as template, 0.8 μL of primer pair sets, 10 μL SYBR® Green Real-time PCR Master Mix (TOYOBO, Japan) and 8.2 μL ddH_2_O. Amplification was performed with pre-denaturation for 60 s at 98 °C, which was then followed by 40 cycles of 98 °C/15 s and 60 °C/60 s. At the end of each reaction, the fluorescence signal was detected and used to generate an amplification profile. For each of the duplicates, quantitative assays were performed in triplicate and a repetitive average *C*t value was taken at no more than 0.5. In this study, the comparative *C*t method was used to express all genes within a sample relative to the time 0 sample. Briefly, the relative *C*t method consisted of the following formula: 2^−(Δ*C*t treatment − Δ*C*t control^), where (*C*t^target gene^ to *C*t^normalizer^) and (*C*t^time zero^ to *C*t^normalizer^) represent Δ*C*t^treatment^ and Δ*C*t^control^, respectively.

**Table 1.** Primer sequences used in this study.

Gene	Forward (5′–3′)	Reverse (5′–3′)
*OsWRKY45*	CGGGTAAAACGATCGAAAGA	GCTGAGACGACACATCAACAA
*JAmy*	TAGGGGTTCAAAGAGGACCA	TCCTCAGTGCAATTCTGGAG
*SalT*	CGAAATAATGTTCCATGGTGTT	TGTACTACGGATCGGTGCAA
*OsPR1b*	ACGGGCGTACGTACTGGCTA	CTCGGTATGGACCGTGAAG
*PBZ1*	GAGCCGCAGAAATGTCCAA	AGGCACATAAACACAACCACAAAC
*OsRAR1*	CTCAAGGTGCCGTCAAGGTT	GCAGGCTTCTCAACAGGTA
*OsSGT1*	GCCATTGAACTTGACCCATC	CAGATGCGAACGAGTAACCC
*OsHSP90*	GTCCCTCATCATCAACACCTTC	TCGGGGACAATGTGGATGAAC

## Results and discussion

It is known that plants respond to microbial pathogen attacks through the activation of a set of defence responses mediated through multiple signalling pathways, including SA, JA, ET and ABA.[17] In this study, the expression patterns of resistance-related genes in true resistance and field resistance response were examined.

### *R*-mediated resistance and the SA- and JA-dependent signalling pathway

**Figure 1.**
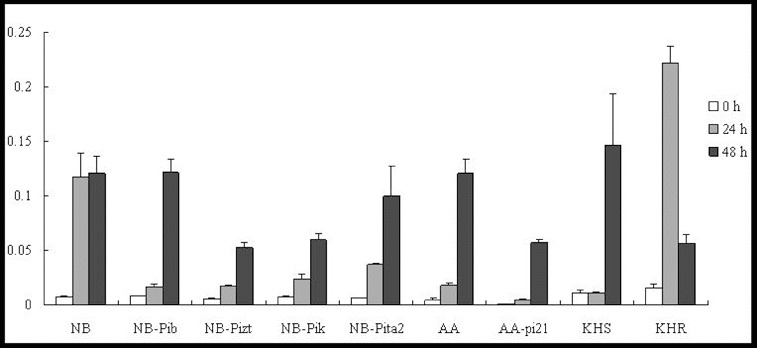
Expression pattern of *OsWRKY45* in the studied rice lines at 0 h, 24 h and 48 h p.i. with *M. oryzae* spores.

*OsWRKY45* is specifically induced by SA and is regarded as a marker gene involved in the SA signalling pathway in defence responses.[10] In our study, the relative expression of this marker gene was analysed by qRT-PCR. The results showed that the *OsWRKY45* gene was induced at 24 h in lines NB, NB-Pib, NB-Pizt, NB-Pik and NB-Pita2. The expression level was shown to increase slightly by 48 h p.i. in the susceptible cultivar NB, which was accompanied by a rapid increase to 15-, 9-, 8- and 16-fold of the background levels in the resistance lines (Figure 1). Further studies with a greater range of time points would be needed to verify these results.

In the field resistance response, *OsWRKY45* was upregulated at 24 h in both cultivars, AA and AA-pi21 (Figure 1). The *OsWRKY45* expression further increased significantly at 48 h and reached a much higher level (63-fold of the background level) in the susceptible AA line than in the resistant AA-pi21 one (about 25-fold of the background level). In KHS, *OsWRKY45* could not be induced at 24 h but could increase 13-fold after 48 h, whereas in KHR, the expression levels increased 14.5-fold after 24 h and 3.7-fold after 48 h (Figure 1). In other words, *OsWRKY45* was upregulated in all of the tested lines, but its levels of expression in the resistant cultivars were much lower than that in the respective susceptible ones. Only in the resistant KHR, the expression level of *WRKY45* was higher than in the susceptible KHS. Thus, it can be speculated that probably only *Pikahei* is involved in the SA signalling pathway.

**Figure 2.**
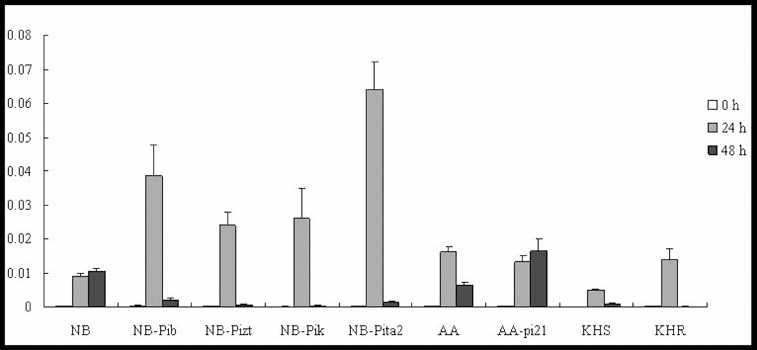
Expression pattern of *JAmyb* in the studied rice lines at 0 h, 24 h and 48 h p.i. with *M. oryzae* spores.

Several studies have suggested that the JA signalling pathway is an essential and important endogenous signal implicated in induced resistance to pathogen infection.[18] In our experiments, the expression patterns of *JAmyb* in the four resistant NB lines (NB-Pib, NB-Pizt, NB-Pik and NB-Pita2) were not similar. They all showed upregulation, but their expression levels differed significantly, being highest in NB-Pita2 (Figure 2). *JAmyb* was upregulated at 24 h, but then its expression decreased by 48 h p.i. In NB, the expression levels were upregulated at 24 h followed by a slight rise at 48 h. The expression levels of the four resistance lines were much higher than those shown in NB. *JAmyb* was induced in two lines with a 62-fold increase at 24 h in AA and a 61-fold increase at 48 h in AA-pi21 (Figure 2). *JAmyb* was also upregulated in KHS and KHR, peaking at 24 h; however, KHR demonstrated slightly higher expression levels than KHS. That is, *JAmyb* showed higher upregulation in resistance lines with NBS-LRR R genes (NB-Pib, NB-Pizt, NB-Pik, NB-Pita2 and KHR with *Pikahei*) than in NB and KHS, suggesting that NBS-LRR R genes may be specifically involved in the JA signalling pathway. Moreover, *JAmyb* was rapidly upregulated by 24 h p.i., but decreased significantly by 48 h p.i., indicating that JA signalling may play an important role in the early stages of the blast fungus infection by causing a downstream defence response.

### Expression profiles of the *SalT* gene: the marker gene of the ABA signalling pathway

**Figure 3.**
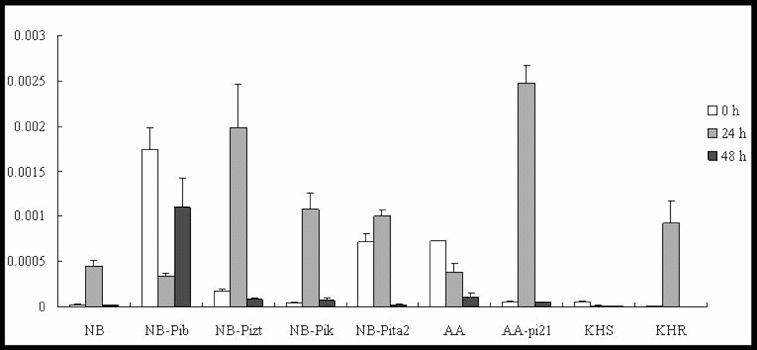
Expression pattern of *SalT* in the studied rice lines at 0 h, 24 h and 48 h p.i. with *M. oryzae* spores.

ABA has also been identified as a key signalling molecule in plant–pathogen interactions, enhancing the susceptibility of rice to blast fungus through the molecular mechanisms behind ABA.[19] ABA treatment has been shown to suppress SAR induction, indicating an antagonistic interaction between SA and ABA signalling.[20] Our results showed differential expression profiles of *SalT*, which is a marker gene for the ABA-signalling pathway, in the resistance response. In the true resistance response, *SalT* was induced and reached a 19-, 12- and 24-fold increase in the expression levels by 24 h p.i. in lines NB, NB-Pizt and NB-Pik, respectively (Figure 3). Only slight upregulation was shown in NB-Pita2 and downregulation in NB-Pib. In the field resistance gene-mediated resistance response, *SalT* was downregulated in the susceptible lines AA and KHS, significantly upregulated (46.5-fold) in AA-pi21 and upregulated 15-fold in KHR at 24 h p.i. (Figure 3). The expression levels of *SalT* did not change and were not downregulated in the tested lines by 48 h p.i. These results suggest the involvement of *SalT* in the *R/Avr* interaction, including *R* genes *Pizt*, *Pik*, *pi21* and *Pikahei*. Moreover, *SalT* may have also negatively regulated *Pib/AvrPib* interaction.

Thus, the differential upregulation of the marker gene *SalT* in all of the tested lines suggested that ABA may be involved in the innate immunity of the rice but under different roles in respective *R* gene-mediated defence responses. There remains much scope for further study in the detection of regulatory factors involved in the crosstalk of ABA with other hormones in plant defence.

### Expression patterns of *OsPR* genes in the presence of *R* gene

Encompassing a variety of novel proteins, PR proteins are known as an essential component in systemic acquired resistance. The accumulation of PR proteins by the induction of defence signalling molecules is a sign of active plant defence. The *PBZ1* gene, also known as *OsPR10*, has been shown to be differentially expressed under most tested conditions.[7,21]

**Figure 4.**
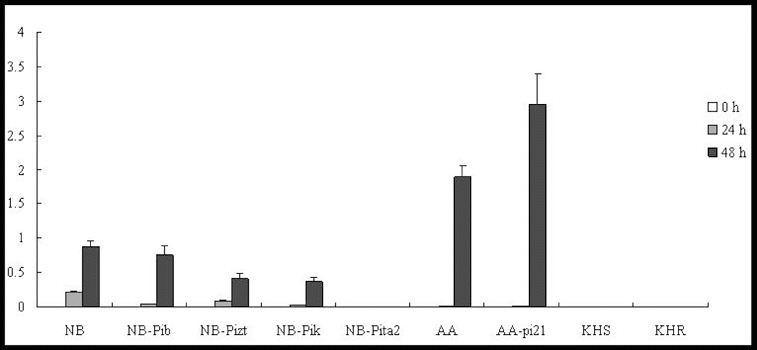
Expression pattern of *OsPR1b* in the studied rice lines at 0 h, 24 h and 48 h p.i. with *M. oryzae* spores.

**Figure 5.**
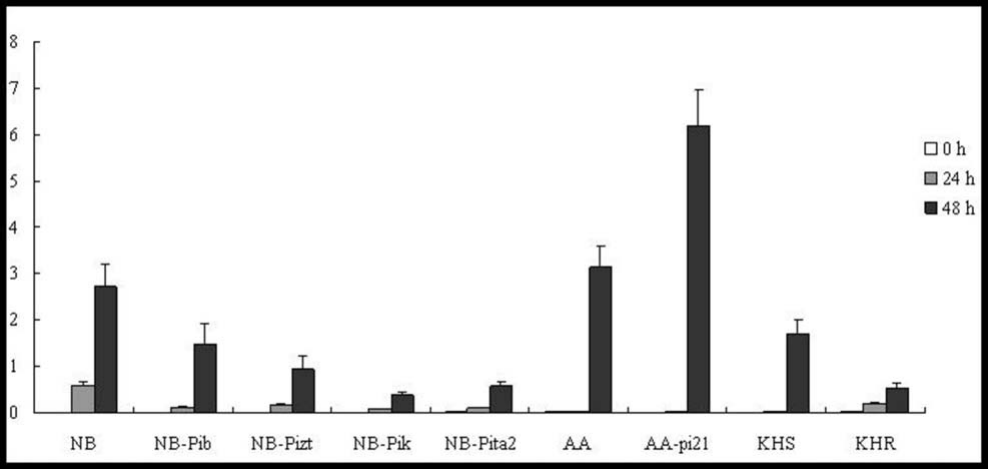
Expression pattern of *PBZ1* in the studied rice lines at 0 h, 24 h and 48 h p.i. with *M. oryzae* spores.

In our study, at the tested time point, *OsPR1b* was shown to be upregulated in four lines (NB, NB-Pib, NB-Pizt and NB-Pik). However, much higher expression levels were shown at 48 h p.i. than at 24 h p.i. No expression was observed in NB-Pita2 at the three time points. *OsPR1b* was significantly induced by 48 h p.i. in AA and AA-pi21; however, the expression level in AA-pi21 was higher than that in AA (Figure 4). Strangely, *OsPR1b* was not expressed in lines KHS and KHR, and an experimental error was ruled out. *PBZ1* was significantly elevated at 24 h in five lines (NB-Pib, NB-Pizt, NB-Pik, NB-Pita2 and AA-pi21), peaking at 48 h at approximately 340-, 155-, 156-, 45- and 35-fold, respectively (Figure 5). Compared with NB, the expression level of *PBZ1* in the resistant lines was lower. The expression profiles of *PBZ1* in AA and AA-pi21 were similar to that of *OsPR1b*. However, *PBZ1* was upregulated with expression levels in KHS higher than those in KHR (Figure 5).

Taken together, the results in Figures 4 and 5 clearly show that the expression levels of *Pib*, *Pizt*, *Pik* and *Pita2* in all NB resistant lines were much lower than the one in the NB-susceptible cultivar; no expression of *OsPR1b* was detected in both KHS and KHR cultivars (Figure 4), and the expression level of *PBZ1* was much lower in KHR than in KHS (Figure 5); the expression level of both genes was higher in the resistant AA-pi21 cultivar, so it can be speculated that only *pi-21* is involved in the control of PR expression. Curiously, *OsPR1b* was not induced in NB-Pita2, KHS and KHR. The exact molecular mechanism behind this, however, warrants further study.

### *OsRAR1*, *OsHSP90* and *OsSGT1*: differentially expressed in *R*-mediated resistance response

*OsRAR1* is an eukaryotic zinc-binding protein that has been associated with *R* gene-triggered innate immune responses to pathogen attack.[22] Previous studies have demonstrated the conserved functions of rice immunity, where OsRAR1 physically interacts with OsSGT1 and OsHSP90 but is not a requirement for all *R* genes.[22–24]

**Figure 6.**
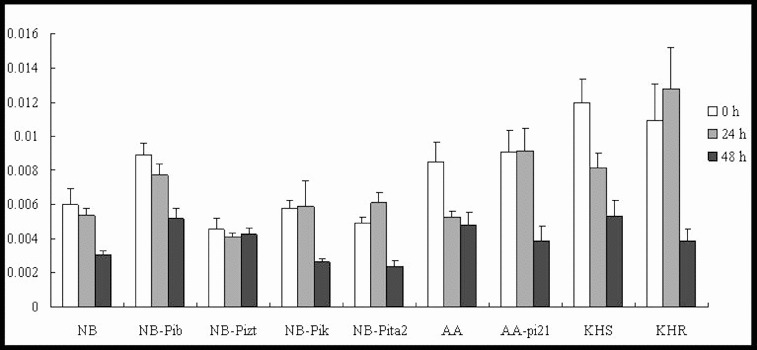
Expression pattern of *OsRAR1* in the studied rice lines at 0 h, 24 h and 48 h p.i. with *M. oryzae* spores.

**Figure 7.**
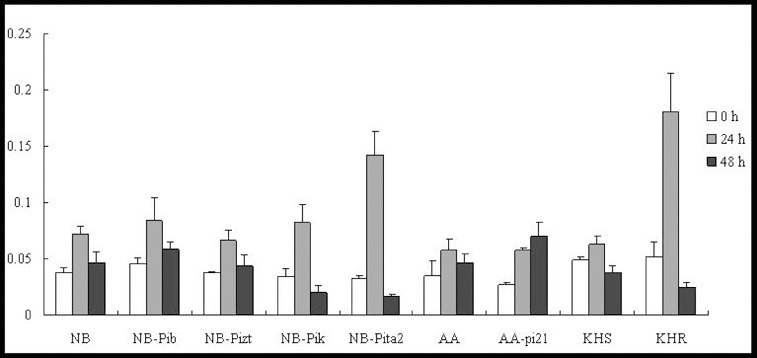
Expression pattern of *OsSGT1* in the studied rice lines at 0 h, 24 h and 48 h p.i. with *M. oryzae* spores.

Our results showed that *OsRAR1* expression levels were downregulated in all of the tested lines, except for NB-Pita2 and KHR (Figure 6). In addition, *OsSGT1* was upregulated in five lines (NB-Pib, NB-Pizt, NB-Pik, NB-Pita2 and KHR) to 2-, 2-, 2-, 2.5- and 4.5-fold of their background by 24 h p.i. (Figure 7). They subsequently decreased rapidly by 48 h p.i in NB, NB-Pib, NB-Pizt, NB-Pik and NB-Pita2. *OsSGT1* was upregulated in two lines: AA and AA-pi21. The expression levels were 1.6-fold at 24 h in AA and 2.6-fold at 48 h in AA-pi21, demonstrating a substantial increase in the resistant line AA-pi21 (Figure 7). *OsSGT1* was induced at 24 h in two lines (KHR and KHS), where the expression level of KHR was much higher than in KHS.

**Figure 8.**
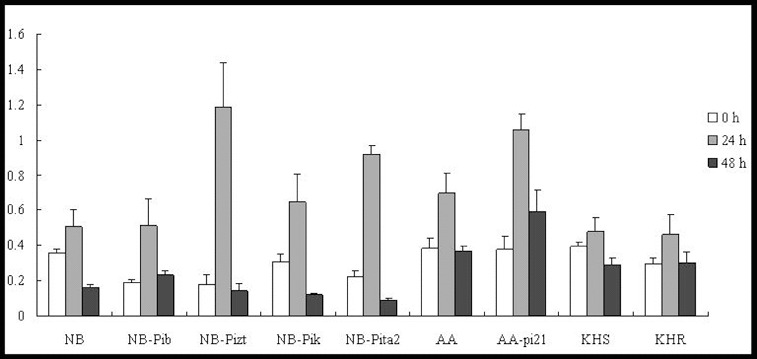
Expression pattern of *OsHSP90* in the studied rice lines at 0 h, 24 h and 48 h p.i. with *M. oryzae* spores.

The expression patterns of *OsHSP90* were similar in all of the tested lines, demonstrating upregulation at 24 h to 1.5-, 2.7-, 6.7-, 2.1 and 4.1-fold in NB, NB-Pib, NB-Pizt, NB-Pik and NB-Pita2, respectively (Figure 8). *OsHSP90* was upregulated to 1.8-, 2.8-, 1.2- and 1.6-fold of the corresponding background levels by 24 h p.i. in AA, AA-pi21, KHS and KHR, respectively. However, the gene was downregulated by 48 h p.i. in all of the tested lines.

The present study demonstrated that *OsRAR1* could only be upregulated in the true resistance line NB-Pita2 and field resistance line KHR. Meanwhile, *OsSGT1* and *OsHSP90* were upregulated in all of the tested lines, suggesting that OsRAR1 was required for *Pita2* and *Pikahei* to interact with *OsSGT1* and *OsHSP90*. In other lines, *OsSGT1* and *OsHSP90* may have also interacted with another unknown protein to provoke a rice immunity response.

## Conclusions

Following a microbial pathogen attack, plants are known to activate a set of defence responses which are mediated through multiple signalling pathways, including SA, JA, ET and ABA, among others. In this study, the expression profiles of innate rice immunity related genes (*WRKY45*, *JAmyb*, *SalT*, *OsPR1b*, *PBZ1*, *OsSGT1* and *OsHSP90*) were examined in the mediated-resistance response of true/field resistance genes. It was shown that the respective resistance genes were involved in specific pathways in addition to common signalling. This study provides important insights into the molecular mechanism behind the resistance response.
